# A mechanism for FtsZ-independent proliferation in *Streptomyces*

**DOI:** 10.1038/s41467-017-01596-z

**Published:** 2017-11-09

**Authors:** Fernando Santos-Beneit, David M. Roberts, Stuart Cantlay, Joseph R. McCormick, Jeff Errington

**Affiliations:** 1 Centre for Bacterial Cell Biology, Medical School, Newcastle University, Newcastle Upon Tyne, NE2 4AX UK; 2 Department of Biological Sciences, Duquesne University, Pittsburgh, PA 15282 USA

## Abstract

The central player in bacterial cell division, FtsZ, is essential in almost all organisms in which it has been tested, with the most notable exception being *Streptomyces*. Streptomycetes differ from many bacteria in growing from the cell tip and undergoing branching, similar to filamentous fungi. Here we show that limited cell damage, either mechanical or enzymatic, leads to near complete destruction of mycelial microcolonies of a *Streptomyces venezuelae ftsZ* mutant. This result is consistent with a lack of *ftsZ*-dependent cross-walls and may be inconsistent with a recently proposed role for membrane structures in the proliferation of *ftsZ* mutants in other *Streptomyces* species. Rare surviving fragments of mycelium, usually around branches, appear to be the preferred sites of resealing. Restoration of growth in hyphal fragments of both wild-type and *ftsZ* mutant hyphae can occur at multiple sites, via branch-like outgrowths containing DivIVA protein at their tips. Thus, our results highlight branching as a means of FtsZ-independent cell proliferation.

## Abstract (web summary)

Protein FtsZ plays key roles in cell division and is essential in most bacterial species; exceptions include streptomycetes, which grow from the cell tip and form branched hyphae. Here, Santos-Beneit *et al*. show that branching allows FtsZ-independent proliferation in *Streptomyces venezuelae*.

## Introduction

Virtually all bacteria possess a peptidoglycan cell wall and proliferate via a conserved division machinery governed by the tubulin-like FtsZ protein^1,2^. The rare exceptions are mainly pathogens or symbionts with greatly reduced genomes (eg, *Mycoplasma* and *Chlamydia*), with a few free living examples (eg, *Planctomyces*). In almost all cases in which it has been examined, the FtsZ system is essential for viability and proliferation of the organism. The two instances where *ftsZ* has turned out to be dispensable are *Mycoplasma* ^3^, which has no cell wall, a reduced genome size and highly specialised life style, and *Streptomyces coelicolor* ^4^, a complex, free living organism, with an apparently conventional division apparatus^5^. We recently showed that *ftsZ* can be deleted in *Escherichia coli*, but only after introduction of one or more compensating mutations that enable the switch to a very bizarre ‘coliflower’ morphology^6^. Actinomycetes such as *Streptomyces* form an unusual branching mycelium in which cross-walls (dependent on FtsZ) form relatively infrequently^7^. FtsZ becomes particularly important when these organisms sporulate. This normally occurs within aerial hyphae, and involves simultaneous formation of a ladder-like array of FtsZ rings, followed by septation to form chains of uninucleate spores^8^. The *ftsZ* mutant turns out to be asporogenous, but nevertheless capable of efficient vegetative growth.

In principle, proliferation–increase in the number of viable cell units–requires the partitioning of a membrane-bound compartment into two compartments (or more), which requires a membrane fission event. Bacterial cells normally maintain a high internal osmotic pressure, which presses outwards on the cytoplasmic membrane, generating turgor. The membrane follows the shape of the cell, which is in turn determined by the peptidoglycan cell wall. The FtsZ machine (divisome) is needed to direct ingrowth of the cell wall, driving membrane fission and septal closure. Loss of cell-wall integrity is normally catastrophic because the turgor pressure causes explosive expansion of the cytoplasmic membrane through the lesion, followed by bursting and loss of cytoplasmic contents into the surrounding medium. Recently, a novel form of cytoplasmic compartmentalisation based on complex membrane invaginations or cross-membranes has been described in *Streptomyces*, and reported to contribute to the proliferation of *ftsZ* mutants^9,10^. However, we think it unlikely that such membrane convolutions could by themselves prevent lysis.

Here we describe the isolation and characterisation of an *ftsZ*-null mutant of *Streptomyces venezuelae*. The mutant is viable, as was observed for the original *Streptomyces coelicolor* mutant^4^. Given that *ftsZ* mutants from many organisms have turned out to be lethal, we wished to learn how *Streptomyces ftsZ* mutant cells were viable. We supposed that streptomycetes either have an alternative mechanism of compartmentalisation, such as the recently described cross-membrane structures^9,10^, or that they are somehow more able than other bacteria to survive mechanical fragmentation and then rebuild and remodel damaged cell membrane and wall structures. It also seemed to us that very little work has been done on survival from, and repair of, mechanical damage in bacteria. Our results suggest that the habit of streptomycetes to form hyphae that grow from the cell tip, together with their ability to form branched structures by generating new tips, contribute to their ability to survive fragmentation and for the fragments to resume growth.

## Results

### Construction of an *ftsZ*-null mutant of *S. venezuelae*

The *ftsZ* genes of both *S. venezuelae* (SVEN_1737) and *S. coelicolor* (SCO2082) are located in highly conserved gene clusters (SVEN_1732-47 and SCO2077-92, respectively) coding for proteins required for efficient growth and branching (*divIVA*), sporulation (*ftsQ*, *ftsW*, *ftsI* and *fstL*) and cell-wall (peptidoglycan) precursor synthesis (*mur* genes). Previously, an *S. coelicolor* Δ*ftsZ* mutant was shown to be viable, although it was blocked in septum formation and sporulation^4^. It seemed likely that *S. venezuelae* would also be able to survive deletion of *ftsZ*. To test this, we attempted to construct an *S. venezuelae* mutant similar to that of the *S. coelicolor* (Supplementary Fig. 1a, b).

On the basis of the construction method that we employed (see ‘Methods’), the *ΔftsZ* mutant should have an Apr^R^ and Kan^S^ phenotype. In initial experiments, all colonies of this genotype that were picked had both wild type and mutant alleles when checked by PCR. Wild-type and mutant chromosomes must frequently exist in syncytial filaments. However, by prolonging the incubation period, and then specifically picking small, poorly growing colonies, *ftsZ* mutant colonies were reproducibly and reliably obtained. Extensive PCR checks confirmed the allelic status of the null mutants. Preliminary microscopic examination revealed that the wild-type and genetically complemented mutant formed abundant aerial hyphae and spore chains, as well as vegetative cross-walls, as expected (Supplementary Fig. 1c, d). However, these structures were absent in the *ftsZ* mutant, although it was nevertheless clearly proficient in mycelium formation with abundant branching hypha. Tip-elongation rates were similar for wild-type (0.10 µm min^−1^ ± 0.01) and mutant (0.08 µm min^−1^ ± 0.01) strains (*n* = 3 for each strain).

**Fig. 1:**
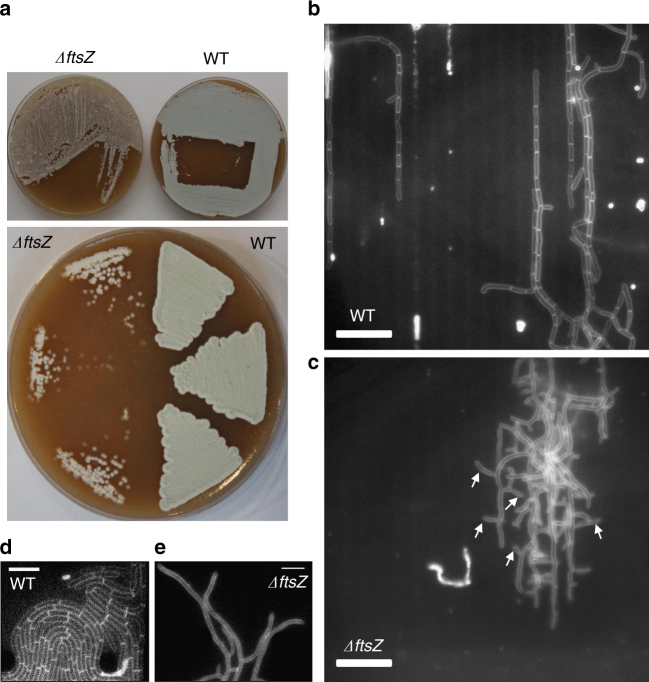
Comparison of growth and cross-wall formation in wild-type and ∆*ftsZ* cells. **a** growth and re-streaking of *ΔftsZ* and wild-type cells on TBO agar plates. Top panel shows growth of the strains from freezer stocks. Bottom panel shows cell regrowth in both mutant and wild-type from three independent colonies following growth at 30 °C for 3 days. **b**, **c** representative images of wild-type (**b**) and *ΔftsZ* mutant (**c**) cells growth in our home-made microfluidic device in the presence of GYM media. Cell membranes were stained with FM4-64 dye. Note the presence of cross-walls in wild-type cells. White arrows indicate cell branching in the *ΔftsZ* mutant. Scale bar = 10 µm. **d**, **e** representative image of wild-type (**d**) and *ΔftsZ* mutant (**e**) cells growth in GYM media in the CellASIC ONIX microfluidic chamber. Cell wall was labelled with 50 µM NADA. Scale bar = 5 µm

Surprisingly, given that the *ftsZ* mutant does not sporulate, and presumably makes no cross-walls to generate separated progeny cells, simply streaking a colony on a fresh plate, by standard microbiological methods, generated hundreds of colony-forming units (Fig. 1a). Given that the mutant does not form spores and the hyphae should not contain cross-walls, it was not obvious how viable fragments of the mutant could arise. This simple observation raised interesting questions about the mechanism of proliferation of the mutant, which we went on to address.

### The *S. venezuelae ftsZ*-null mutant lacks visible cross-walls

*Streptomyces* spore preparations are frequently used to provide an efficient and reproducible starting inoculum for batch cultures or genetic crosses. However, it is also possible to propagate streptomycetes from vegetative mycelia, and this route is essential for analysis of non-sporulating mutants. In this case, propagation presumably involves fragmentation of mycelium, followed somehow by recovery and growth of the fragments. Little, if any, research seems to have been done on how fragmentation and regrowth occur. We began to examine this by checking that the *ftsZ* mutant is indeed defective in cross-wall formation. For these and subsequent experiments, we took a sample from a glycerol stock of the mutant, mechanically fragmented the material by multiple passages through a pipette tip and then incubated the fragments in liquid growth medium for one hour before starting the imaging. WT and *ΔftsZ* mutant cultures were then imaged within two different microfluidic devices. Figure 1b and c shows typical images of small segments of wt or *ΔftsZ* mutant mycelia stained for membrane with FM4-64 dye in a microchannel device^11^. Under these conditions, the *Streptomyces* mycelia are partially constrained in narrow (~1 µm) agarose channels (oriented vertically in the figure, but not visible in the fluorescence images), but hyphal branches can escape and grow into the space between agar and glass. Arrows in Fig. 1c point to the examples of branches made by the *ftsZ* mutant that appear typical of *S. venezuelae*. However, as expected, cross-walls were only visible in the wild-type mycelium.

In another series of experiments, we imaged cells in a CellASIC ONIX microfluidic system, which enables the imaging of cells under well-controlled environmental conditions, but fewer geometrical constraints^12^. In this case, the cells were incubated with the cell-wall label, NADA^13^, which labels newly synthesised peptidoglycan material. These experiments again revealed the presence of abundant cross-walls in wild-type cells (Fig. 1d), but not in the *ΔftsZ* mutant (Fig. 1e). In many similar experiments under various imaging and growth conditions, we did not detect cross-walls in the *ftsZ* mutant strain (Supplementary Movies 1–3).

**Fig. 2:**
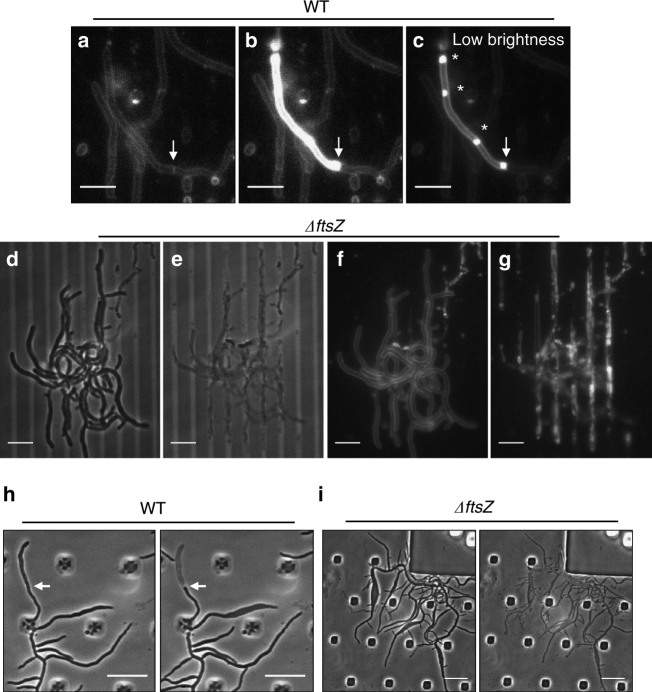
Lack of compartmentalisation in the *ΔftsZ* mutant. **a**–**c** Panels show FM4-64 membrane-stained wild-type cells grown in GYM media in a home-made microfluidic device. Panels **b** and **c** are equivalent, but with lower brightness in the latter. White arrow indicates the cell division site. Yellow asterisks show possible membrane accumulations. Scale bars = 5 µm. **d**–**g** Panels show bright field (**d**, **e**) and FM4-64 stained membrane (**f**, **g**) of the Δ*ftsZ* mutant grown in GYM in a home-made microfluidic device. Scale bars = 5 µm. **h**, **i** growth of wild-type (**h**) and Δ*ftsZ* mutant (**i**) cells in the CellASIC ONIX microfluidic chamber in TSB media with constant lysozyme flow up to 1000 µg ml^−1^. White arrows indicate the presumed presence of a division site. Scale bar = 5 µm

If compartmentalisation of the *Streptomyces* mycelium (whether through conventional cross-walls or any other putative structure) was dependent on FtsZ, then the *ftsZ* mutant mycelium should behave as a single compartment. Strong evidence in favour of this idea came from microchannel experiments similar to those described above (Fig. 2a–g). We noticed that lytic events occasionally occur, presumably due to the combination of cell growth and torsional constraint. A typical example of the behaviour of the *ftsZ* mutant under these conditions is shown in Fig. 2d–g. Figure 2d and e shows two frames from Supplementary Movie 4, separated by 60 min. The whole microcolony lysed in less than an hour (note that the phase dark objects in the middle of the panel that do not change are probably fragments of dead cells from the original loading). Figure 2f and g shows the rapid disintegration of membrane structure during this event (Supplementary Movie 5; see also Supplementary Movies 6 and 7 for an additional example). In contrast to this virtually complete lysis for the mutant, lytic events in the wild-type strain were always limited to relatively small compartments. Figure 2a–c shows a typical lysis event, showing sequential frames, taken from Supplementary Movie 8. Lysis was often accompanied by a marked increase in membrane staining, possibly because efflux pumps that would remove the dye cease to operate or that the dye can access both leaflets of the membrane. In contrast to the *ftsZ* mutant, this kind of lytic event was usually well contained in the wild type and in Fig. 2a and b the arrow points to a cross-wall that appears to prevent lysis of the upper compartment from spreading. The non-lysed compartment below the cross-wall remained viable and exhibited continued growth (Supplementary Movie 8).

### Are cross-membrane structures associated with cell death?

In passing we note that, in general, we had difficulty in detecting the FtsZ-independent cross-membrane structures that have been reported elsewhere^9,10^. The only phenomena we noted that were reminiscent of cross-membranes are illustrated well in Fig. 2a–c. Figure 2c is the same image as Fig. 2b, but with the brightness reduced. Membrane accumulations similar to the reported cross-membrane structures were evident at several places in the bright compartment (asterisks). Many similar events were detected in our experiments (eg, Supplementary Fig. 2a, b), and because the invaginations were not visible before lysis, we believe that they are a product of cell death, rather than functionally useful structures generated by living cells. We have not detected this kind of structure in clearly visible hyphal segments without signs of lysis (eg, Supplementary Movies 4–11; Supplementary Fig. 3a, b).

To exclude the possibility that *S. venezuelae* is unusual in lacking cross-membranes, we treated an *S. coelicolor* wild-type strain with FM4-64 or FM5-95 dyes under two different growth conditions: (a) TSB medium in a microfluidic device and, (b) GYM medium in flasks. Just as for *S. venezuelae*, we only detected cross-membrane like structures in cells that appeared to have shrunk abruptly, presumably by some kind of membrane fracture or other lytic event (Supplementary Movies 12 and 13; Supplementary Fig. 4a, b).

### Deletion of *ftsZ* abolishes mycelial compartmentalisation

To look for compartmentalising structures in larger mycelial masses, we again turned to the ONIX system. Wild-type and *ftsZ* mutant cultures were imaged by phase-contrast time-lapse microscopy. After a period of growth sufficient to allow both cultures to form mycelial microcolonies, lysozyme (1000 µg ml^−1^) was introduced into the chamber to induce lysis. In the wild-type, mycelium lysis always clearly occurred differentially in relatively small discrete compartments of the mycelium (10 movies analysed in detail). The arrow in Fig. 2h points to a presumptive cross-wall that limited lysis to the tip compartment in a typical experiment (see also Supplementary Movies 14 and 15). However, for the *ftsZ*-null mutant, lysis usually occurred in a single event in which the whole mycelial structure went from phase dark to phase pale within one 5 min interval (Fig. 2I; Supplementary Movie 16). In each of the seven experiments with lysozyme at 1000 µg ml^−1^ and three at 100 µg ml^−1^, lysis occurred throughout the mycelium of the *ftsZ* mutant in a single step, with no sign of compartmentalised survival (example with 100 µg ml^−1^ lysozyme in Supplementary Movie 17). Total lytic events were also observed in 7 out of 10 movies analysed for the mutant strain when lysozyme was added at 10 µg ml^−1^ (Supplementary Fig. 5a; Supplementary Movies 18 and 19). In the remaining events, the whole colony stopped growing, as if turgor was lost, but it remained phase dark for reasons that are not clear. In summary, for the wild type, lysis was typically restricted to relatively small segments of mycelium, whereas lysis of the *ftsZ*-null mutant usually occurred in a single event in which the whole mycelial structure went from phase dark to phase pale within a short period of time. Therefore, lytic events resulted in a much greater loss of viable biomass in the *ftsZ*-null mutant than in the wild type. The results suggest that *ftsZ* is absolutely required not only for sporulation, but also for functional compartmentalisation of *S. venezuelae* vegetative hyphae.

### Δ*ftsZ* mycelial fragments may survive by resealing at branch points

**Fig. 3:**
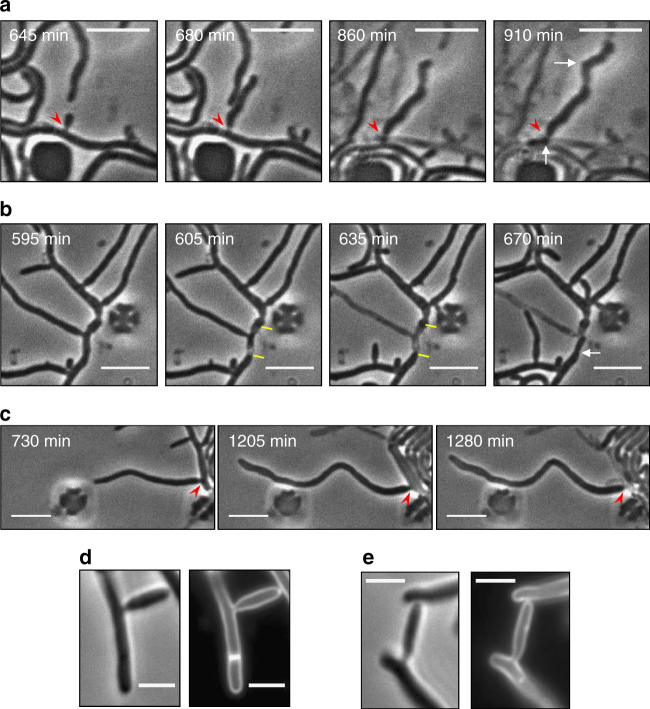
Survival and sealing seem to involve branch points. Effects of lysozyme pulsing on the *ΔftsZ* mutant (**a**) and wild-type (**b**, **c**) cells in the CellASIC ONIX microfluidic chamber. Cells were grown with TSB media. Red arrowheads indicate branch sites; yellow lines highlight presumable cell division sites; and white arrows show sites of cell regrowth. Scale bars = 5 µm. **d**, **e** Brightfield (left panels) and FM4-64 stained (right panels) of wild-type (**d**) and *ΔftsZ* mutant (**e**) cells. Scale bar = 5 µm

If growth of the *ftsZ*-null mutant only produces a single-cell compartment, how does mechanical fragmentation (by microbiological loop or hydrostatic forces in a micropipette tip) generate increased viable cell number? We tested various experimental approaches to visualise rare fragmentation and regrowth events. Eventually, we were able to follow some of these rare events by using a modified version of the lysozyme experiments described above. Instead of applying the lysozyme continuously, we varied the concentration and pulsed the supply on and off until some *ftsZ* mycelia showed incomplete lysis followed by a degree of regrowth (Supplementary Fig. 5b). In most cases, it was difficult to see exactly how the survival and regrowth occurred because the events occurred within the crowded sections of mycelium, but one of the clearer examples is shown as a series of time-lapse frames in Fig. 3a (Supplementary Movies 20 and 21). The red arrowhead points to the branch site at the base of the surviving fragment (see also below), and arrows in the final frame of the sequence point to regions of new hyphal growth, demonstrating that this was a surviving viable fragment. In those cases where the nature of the surviving fragment could clearly be seen, it appeared that the surviving fragment was bounded by a tip and a branch site (Supplementary Movies 22–26). This was not surprising, given that the tip represents a natural closure of the cell cylinder and the other end is normally narrower than the rest of the cell cylinder (see next).

Importantly, compartmentalised survival involving branch points (rather than cross-walls) was also observed in the wild type. Figure 3b shows a time-lapse sequence from an experiment with the wild type, in which lysis occurred in a segment of hypha containing a branch. Hyphal lysis was apparently limited by cross-walls on either side of the branch, indicated by yellow lines (Supplementary Movie 27; see also Supplementary Movies 28 and 29). Figure 3c shows another common event in which lysis apparently occurred in the hypha underlying a branch (marked by the red arrowhead), but the long branch to tip segment remained viable and continued to grow (Supplementary Movie 30; see also Supplementary Movie 31). This last finding raised the intriguing possibility that branch points offer a non-divisomal mechanism for hyphal sealing and protection from mechanical fragmentation.

In support of this idea, we noticed that in *S. venezuelae*, the hyphae tend to narrow at branch sites. Typical branch sites of wild-type and *ftsZ* mutant mycelia, imaged by a fluorescent membrane dye, FM4-64, are shown in Fig. 3d and e (see also several branch points in Fig. 1 and in Supplementary Figs. 2 and 3). On the basis of fluorescence imaging of membrane-stained samples, the branches (wt, 0.32 ± 0.04 µm; mutant, 0.33 ± 0.06 µm) were approximately half the diameter of mature hyphae (wt, 0.65 ± 0.12 µm; mutant, 0.67 ± 0.10 µm). Prototypical branches of *S. venezuelae* are shown in scanning electron microscopic images in Supplementary Fig. 6. We suggest that branch sites provide a preferred site for resealing of hyphal fragments after mechanical fragmentation, either because they fracture to give broken ends that are less ‘ragged’, and/or are narrower, and thus more readily support resealing of the cytoplasmic membrane and wall regeneration. It is also possible that proteins involved in branch-site elaboration render the branch less susceptible to lysis.

### Δ*ftsZ* mycelial fragments recover via tip growth and *de novo* branching

To improve our understanding of how *ftsZ*-null mutant fragments recover and resume growth, the fate of 632 fragments (followed in a series of 96 movies) was analysed by time-lapse microscopy and the ONIX device. The design of the system filters cells according to size, so we were able to select out a population of small cell fragments (typically 5–10 µm) that were easier to quantify. Of the 632 fragments, 128 (20%) showed significant growth within 12 h of incubation. Of the fragments that were able to grow, 61 (48%) re-started growth from one or both poles, 35 (27%) regrew from multiple sites (including one or both poles), 26 (20%) showed more complex patterns such as bulging, followed by branching and 6 (5%) began growing, but then lysed soon after. Supplementary Fig. 7a and b shows typical examples of fragment regrowth of the mutant (see also Supplementary Movies 32–34 for examples of the different patterns of growth). In panel Supplementary Fig. 7a, regrowth occurred from both tips of the fragment. In both cases, the base point from which new growth occurred (arrowheads) appeared to be narrow suggesting that it was generated by a branch-like fragmentation mechanism. In panel Supplementary Fig. 7b, regrowth was initiated at multiple sites on the fragment but again the new growth sites appeared to start from branch-like constrictions. Similar observations (42% re-starting growth from the poles, 33% from multiple sites and 25% showing complex patterns or premature lysis) were made for wild-type fragments (*n* = 80) (although the percentage of fragment regrowth was higher, 41%, in this strain). Two typical examples for wild type are shown in Supplementary Fig. 7c and d (see also Supplementary Movie 35 for an undefined pattern). Thus, new growth of wild-type and *ftsZ*-null mutant fragments can occur from previously active tips or from other sites, apparently including new branches.

### DivIVA associates with growth sites in recovering fragments

**Fig. 4:**
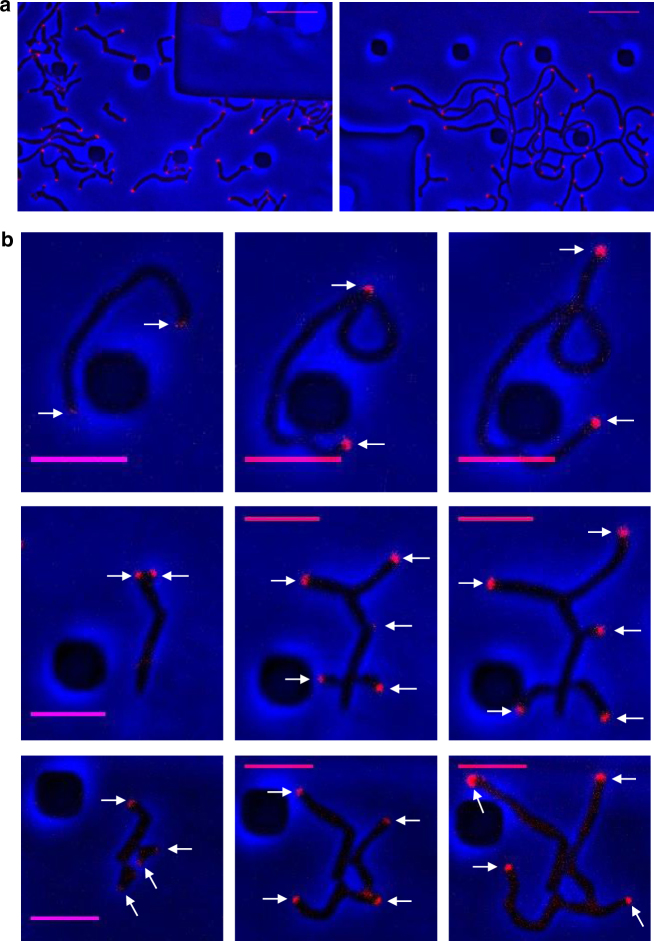
Cell regrowth following fragmentation. **a** DivIVA tip localisation in wild type (left panel) and Δ*ftsZ* mutant (right panel). **b** Representative examples of the types of cell regrowth of fragments in the Δ*ftsZ* mutant from both ends (top), multiple/new sites (middle) and from an existing pole and/or bulge (bottom). All spores and fragments were grown in the CellASIC ONIX microfluidic chamber with TSB medium, using a 500 ms exposure for acquiring the mCherry signal. White arrows indicate the sites of DivIVA-mCherry localisation. Scale bars = 10 µm in **a** and 5 µm in **b**

Following the observation that regrowth can occur at various places within the fragment (at cell poles or via de novo branching), it was predicted that this process was mediated by the presence of DivIVA at these sites, essential for driving the formation of the tip-elongation complex. To test this, the gene expressing DivIVA-mCherry fusion protein was introduced into both wild-type and *ΔftsZ* mutant strains. Fragments of *ΔftsZ* and wild-type spores were loaded into the ONIX device and, as expected, DivIVA-mCherry was localised at the tips and nascent branch sites during the re-establishment of fragment growth and during spore germination, respectively (Fig. 4; Supplementary Movies 36–40). The *ΔftsZ* strain expressing DivIVA-mCherry was further subjected to a lysozyme pulsing experiment as described above, and again polar DivIVA-mCherry was observed during cell growth and branching. Upon lysis of the majority of the mycelial mass, the surviving fragment (presumably corresponding to a surviving branch) retained DivIVA-mCherry signal at the poles and branch sites, before growth was re-established, leading to a new mycelial mass (Supplementary Movie 41). These data indicate that upon cell fragmentation or branch-site survival, DivIVA is either recruited to poles and branch sites in non-growing fragments or is retained at these regions in actively growing cells, providing a means for continued cell viability and proliferation in the absence of divisome-mediated cell division.

## Discussion

We isolated an *S. venezuelae ftsZ*-null mutant equivalent to the *S. coelicolor* mutant that was described 20 years ago^4^ and demonstrated that it too is viable, suggesting that this is a general property of the streptomycetes. Like the original mutant, this one was also able to grow vegetatively, but not form spores. On the basis of many experiments done in the course of generating the results described above, we can conclude that the cross-walls, if made at all by the mutant, must be extremely rare.

**Fig. 5:**
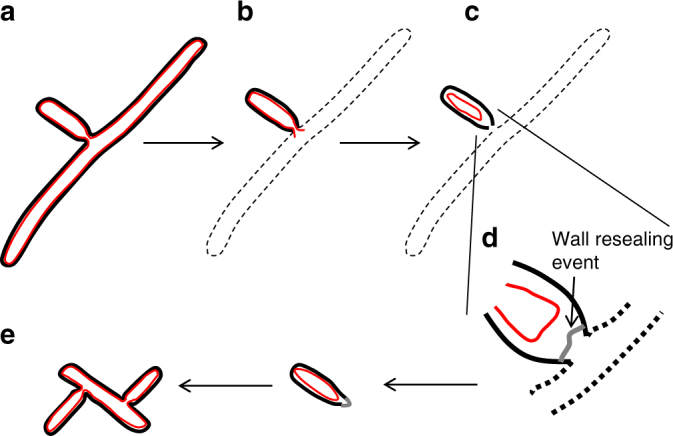
Model for survival and resumption of growth of *ΔftsZ* mycelial fragments. A growing branch (**a**) is attached to an underlying hyphal segment that undergoes lysis (**b**). Some of the contents of the branch are spilled, but membrane retraction and resealing results in survival of the branch (**c**). Presumably, the peptidoglycan wall is initially punctured, but it may be that the narrow diameter at the branch-site limits the amount of wall repair that is needed to enable restoration of turgor (**d**). Finally, the sealed segment resumes growth by formation of one or more de novo branches on its surface (**e**). Black lines represent the cell wall; red lines represent the cell membrane. Note that this would be a rare event and most mycelial fragments of the *ftsZ* mutant are destroyed by mechanical damage

A possible series of steps required for fragmentation propagation of the *ftsZ* mutant, which have emerged from our work, are illustrated in Fig. 5. Briefly, we imagine that survival of a segment of mutant hypha (Fig. 5b) will depend on retention of sufficient cell cytoplasmic components needed to eventually restart growth, together with spontaneous resealing of the membrane (red line) at the broken end (Fig. 5c). The cell then presumably needs to repair (grey) the peptidoglycan wall (black) outside the membrane in order to restore turgor (Fig. 5d). Finally, a mechanism is required to enable the resumption of growth (Fig. 5e).

In the *ftsZ* mutant that lacks cross-walls, the first step (Fig. 5b) is presumably an inefficient event that may be highly dependent on how far the segment is from the immediate site of enzymatic or mechanical damage. The high cellular turgor pressure will tend to result in expulsion of cell contents at the site of damage. We noticed that the survival and regrowth events tended to occur within large mycelial masses, so it is possible that survival is promoted by twisting or compression of cell membrane within hyphae, thereby limiting the outflow of cytoplasmic components. Of course, in the wild type this would be much less important because cross-walls will efficiently confine lysis to a single small compartment.

In some relatively ‘sheltered’ parts of the mycelium, the osmotically driven flow of cytoplasm may reduce sufficiently for membrane resealing to occur. It is generally thought that lipid bilayers should spontaneously reseal (Fig. 5c) on thermodynamic grounds. However, in principle, the efficiency of resealing will also depend on the size of the lesion. This could explain why the tip distal ends of surviving fragments tended to coincide with branch points, which are narrower than the rest of the hyphae. The ability of narrow broken segments of wall to survive is reminiscent of the ‘coliflower’ mutants of *E. coli*, which can also proliferate in the absence of a functional divisome. In these mutants, the ability to survive seems to be dependent on relaxation of the normal tight control over cell cylindrical diameter, enabling the formation of narrow necks between bulbous cells, which can spontaneously fracture and then apparently reseal.

Next, the resumption of growth presumably requires some form of cell-wall repair (Fig. 5d) so that turgor can be re-established, but how bacterial cells spatially control cell-wall synthesis is poorly understood, even for normal cell extension, never mind for repair synthesis. *Streptomyces* may provide an important new model for investigating this problem.

Finally, after survival, resealing and wall repair, it appears that the fragments frequently resume growth by a branch-like mechanism, often with multiple separate branches being formed (Fig. 5e). The regrowth and de novo branching appeared to be associated with the normal DivIVA-dependent tip growth and initiation apparatus (Fig. 4)^7,14^. Together with the specialised morphology of the branch site, the unusual ability to generate branches *de novo* from lateral walls, may provide *Streptomyces* with another adaptation enabling it to survive fragmentation.

In the light of these observations, branch formation may be seen as an important and previously unrecognised non-divisome dependent mechanism for cell proliferation (increase in cell number) in streptomycetes and the wider mycelial actinobacteria. Two previous reports described membranous structures, or cross-membranes, that occurred frequently, but at rather irregular intervals in mycelia^9,10^. We have also frequently observed similar structures, but our data provide no support that they are involved actively in compartmentalisation, and instead we provide direct evidence that such structures could arise during cell death.

Although killing by antibiotic action has been the subject of significant amounts of research, relatively little seems to be known about killing and survival of bacteria following mechanical fragmentation, and further work is needed to understand fully what happens when cells are fractured and how they might recover. Whatever the mechanism of *ftsZ*-independent proliferation, the ability to grow *Streptomyces* completely devoid of a functional division machine may also facilitate mechanistic studies of the divisome, or even reconstruction of heterologous division machines in the *Streptomyces* host.

## Methods

### Bacterial strains, constructs and growth conditions

See Table 1 for all the strains and plasmids used in this work. The wild-type *S. venezuelae* strain was ATCC10712. The wild-type *S. coelicolor* strain was M145. For the construction of the *S. venezuelae* Δ*ftsZ* mutant (ΔSVEN_1737), the primers SVftsZ59 (TGATGTTGGCCTCGGGGTGGGCGGCCTCGCTGACCAGCTGTGTAGGCTGGAGCTGCTTC) and SVftsZ60 (GCGGCGCGAACCAACGCGCGGCGACGACACGTAACTCGAGATTCCGGGGATCCGTCGACC) were used to amplify the *aac(3)IV* apramycin resistance cassette from pIJ773^15^ with flanking homology to the SVEN_1737 locus (Supplementary Fig. 1). The resulting disruption cassette was introduced into the *S. venezuelae* cosmid, Sv-4-G01, by λ RED-mediated recombination^16^ to create mutagenic cosmid pJK1 in which 844 bp, starting 16 bp upstream of the SVEN_1737 start to codon up, and including codon 276 were replaced with an *apra*-*oriT* cassette. This mutagenic cosmid was introduced into *S. venezuelae* by interspecies conjugation and marked null mutants generated by double homologous recombination events. The mutants were first identified by their apramycin-resistant and kanamycin-sensitive phenotypes, and then confirmed by PCR analyses. For the PCR analyses, different combinations of primers were used either by hybridising within *ftsZ* or within the apramycin resistance *aac(3)IV* cassette, as well as hybridising upstream and downstream of *ftsZ*. A representative isolate was designated DU669. For the complementation studies a 3906 bp *Bam*HI–*Pst*I fragment from Sv-4-G01 was cloned into pMS82^15^ to create pJS8, which was introduced into *S. venezuelae* Δ*ftsZ* mutant strain by conjugation and integrated in trans into the chromosome at the ΦBT1 attachment site (Supplementary Fig. 1). A representative isolate was designated DU670. As control, the pMS82 vector was also introduced into *S. venezuelae* Δ*ftsZ* mutant. A representative isolate was designated DU671.

**Table 1:** Strains and plasmids used in this work

	**Description**	**References**
*Bacterial strains*		
*E. coli* DH5α	F′Ф80 *lacZ∆M15*	^18^
*E. coli* BW25113 (pIJ790)	K-12 derivative (∆*araBAD*, ∆*rhaBAD*) carrying plasmid pIJ790	^19^
*E. coli* ET12567 (pUZ8002)	*dam*, *dcm*, *hsdS*, *cat*, *tet*, carrying helper plasmid pUZ8002	^20^
*S. venezuelae* ATCC10712	Parental strain	ATCC
*S. venezuelae* DU669	*S. venezuelae* Δ*ftsZ*::*aac(3)IV*	This work
*S. venezuelae* DU670	*S. venezuelae* Δ*ftsZ*::*aac(3)IV*/pJS8 (pMS82-*ftsZ* ^+^)	This work
*S. venezuelae* DU671	*S. venezuelae* Δ*ftsZ*::*aac(3)IV*/pMS82	This work
*S. venezuelae* DU786	*S. venezuelae* Δ*ftsZ*::*aac(3)IV*/ pSS204 (*divIVA-mCherry*)	This work
*S. venezuelae* DU787	*S. venezuelae* ATCC10712/ pSS204 (*divIVA-mCherry*)	This work
*S. coelicolor* M145	Plasmid-free derivative of the wild-type *S. coelicolor* A3(2) strain	^17^
*Plasmids or cosmids*		
pIJ773	Vector containing *aac(3)IV*-oriT	^16^
Sv-4-G01/∆ftsZ::aac(3)IV-oriT	*S. venezuelae* cosmid deleted in *ftsZ*	This work
pMS82	Hygromycin integration vector	^15^
pJS8	*ftsZ* with its own promoter into pMS82	This work
pSS204	Vector containing *divIVA-mCherry* fusion	^12^

For the localisation assay of DivIVA, we introduced plasmid pSS204, containing *divIVA*-*mCherry* (Table 1), into *S. venezuelae* Δ*ftsZ* mutant and wild-type strains; obtaining DU786 (Δ*ftsZ*) and DU787 (WT), respectively.

MYM (malt extract, yeast extract, maltose)^17^ was used for conjugation and screening of *S. venezuelae* strains. Routinely 25 µg ml^−1^ of apramycin and kanamycin was used for selection of the strains. Overall, 25 µg ml^−1^ hygromycin was used for pMS82-derived complementation strains. *S. venezuelae* strains were grown on TBO medium (tomato puree 20 g l^−1^; oat meal 20 g L^−1^; agar 25 g L^−1^; pH 6.5) to make glycerol stocks of both *ΔftsZ* non-sporulate cells and WT spores. For analysis of spore formation impression cover slip lifts were made and analysed by phase-contrast microscopy using an Eclipse 400 microscope (Nikon) equipped with a ×100 NA 1.25 phase-contrast objective (Nikon) and a Micropublisher 5.0 RTV camera (Q Imaging). For the microfluidic experiments, cells were grown in either Tryptone Soya Broth (TSB, Oxoid), Difco Nutrient Broth (BD) or GYM (yeast extract 4 g l^−1^; malt extract 10 g l^−1^; glucose 4 g l^−1^; pH 7). For liquid cultures, baffled flasks were inoculated directly using the glycerol stocks containing either fragments or spores. For mechanical disruption, a sample from a glycerol stock of the mutant was mechanically fragmented by multiple passages through a pipette tip and then fragments were incubated in liquid growth medium for one hour before starting the imaging.

### Time-lapse and fluorescence microscopy

Following fragmentation of *ΔftsZ* cells or the preparation of wild-type spores, 75 µl was loaded into B04A microfluidic plates (ONIX, CellASIC) at 4 psi for 30 s, followed by a channel washing step at 3 psi for 30 s. In general, media flow rate was maintained at 2 psi and the temperature at 32 °C for growth of *Streptomyces*. Media switching and flow rate settings were controlled using the CellASIC ONIX FG Software (v 5.5.1.0). For peptidoglycan (cell wall) staining, the media was supplemented with 50 µM NBD-amino-d-alanine (NADA)^13^, and for membrane staining, the media was supplemented with 0.5 µg ml^−1^ FM4-64 (Molecular Probes) or FM5-95 (Invitrogen). For the cell-wall staining time-lapse experiments, cells were grown using the CellASIC ONIX microfluidic chamber and imaged in the presence of GYM, TSB or Difco Nutrient Broth. For membrane staining time-lapse experiments, an agarose-pad based microfluidic device was used as described in Eland et al.,^11^ using GYM and TSB media. For the DivIVA localisation assay cells were grown using the CellASIC ONIX microfluidic chamber and imaged in the presence of TSB medium.

For continuous lysozyme flow experiments, an increasing concentration of lysozyme (0, 1, 10, 100 and 1000 µg ml^−1^ final) was continually supplied to the cells. For lysozyme pulsing experiments, four pulses in total were rapidly applied to the cells for 20 mins at 10 psi. The first two pulses contained lysozyme at low (10 µg ml^−1^) concentration, followed by a further two pulses at higher lysozyme concentration (100 µg ml^−1^). In between pulsing, the cells were grown with lysozyme-free medium at 2 psi.

In all cases, images were acquired using a Nikon Ti microscope equipped with a Nikon Plan Apo ×100/1.4 oil objective and FRAP-AI v. 7.7.5.0 software (MAG Biosystems, Molecular Devices). The images were acquired every 5–15 mins and all images and movies were processed using ImageJ (NIH). Tip-elongation rates were calculated following three different growing tip of both WT and *ΔftsZ* cells during 250 min using ImageJ software. Typical branch sizes of WT and *ΔftsZ* mutant mycelia were calculated using fluorescence imaging of membrane-stained samples (10 examples of each class measured) and ImageJ software.

### Scanning electron microscopy

Scanning electron microscopy (SEM) was performed using a VEGA3 (TESCAN) Scanning Electron Microscope from 4-day-old TSA plates (30 °C). Before the SEM analysis, glutaraldehyde-fixed samples were washed in phosphate buffered saline, dehydrated through a graded ethanol series and critical-point dried with carbon dioxide. Finally, samples were mounted on aluminium stubs and coated with a gold sputter coater.

### Data availability

All relevant data supporting the findings of the study are available from the corresponding author.

## Electronic supplementary material

Supplementary Information

Description of Additional Supplementary Files

Supplementary Movie 1

Supplementary Movie 2

Supplementary Movie 3

Supplementary Movie 4

Supplementary Movie 5

Supplementary Movie 6

Supplementary Movie 7

Supplementary Movie 8

Supplementary Movie 9

Supplementary Movie 10

Supplementary Movie 11

Supplementary Movie 12

Supplementary Movie 13

Supplementary Movie 14

Supplementary Movie 15

Supplementary Movie 16

Supplementary Movie 17

Supplementary Movie 18

Supplementary Movie 19

Supplementary Movie 20

Supplementary Movie 21

Supplementary Movie 22

Supplementary Movie 23

Supplementary Movie 24

Supplementary Movie 25

Supplementary Movie 26

Supplementary Movie 27

Supplementary Movie 28

Supplementary Movie 29

Supplementary Movie 30

Supplementary Movie 31

Supplementary Movie 32

Supplementary Movie 33

Supplementary Movie 34

Supplementary Movie 35

Supplementary Movie 36

Supplementary Movie 37

Supplementary Movie 38

Supplementary Movie 39

Supplementary Movie 40

Supplementary Movie 41
