# Supplementary material for: A mechanism for FtsZ-independent proliferation in Streptomyces
Source: Nat Commun. 2017 Nov 9;8:1378. doi: 10.1038/s41467-017-01596-z (PMC5680176; doi:10.1038/s41467-017-01596-z)
Supplement: Supplementary file 1 — Supplementary Information [file 41467_2017_1596_MOESM1_ESM.pdf]

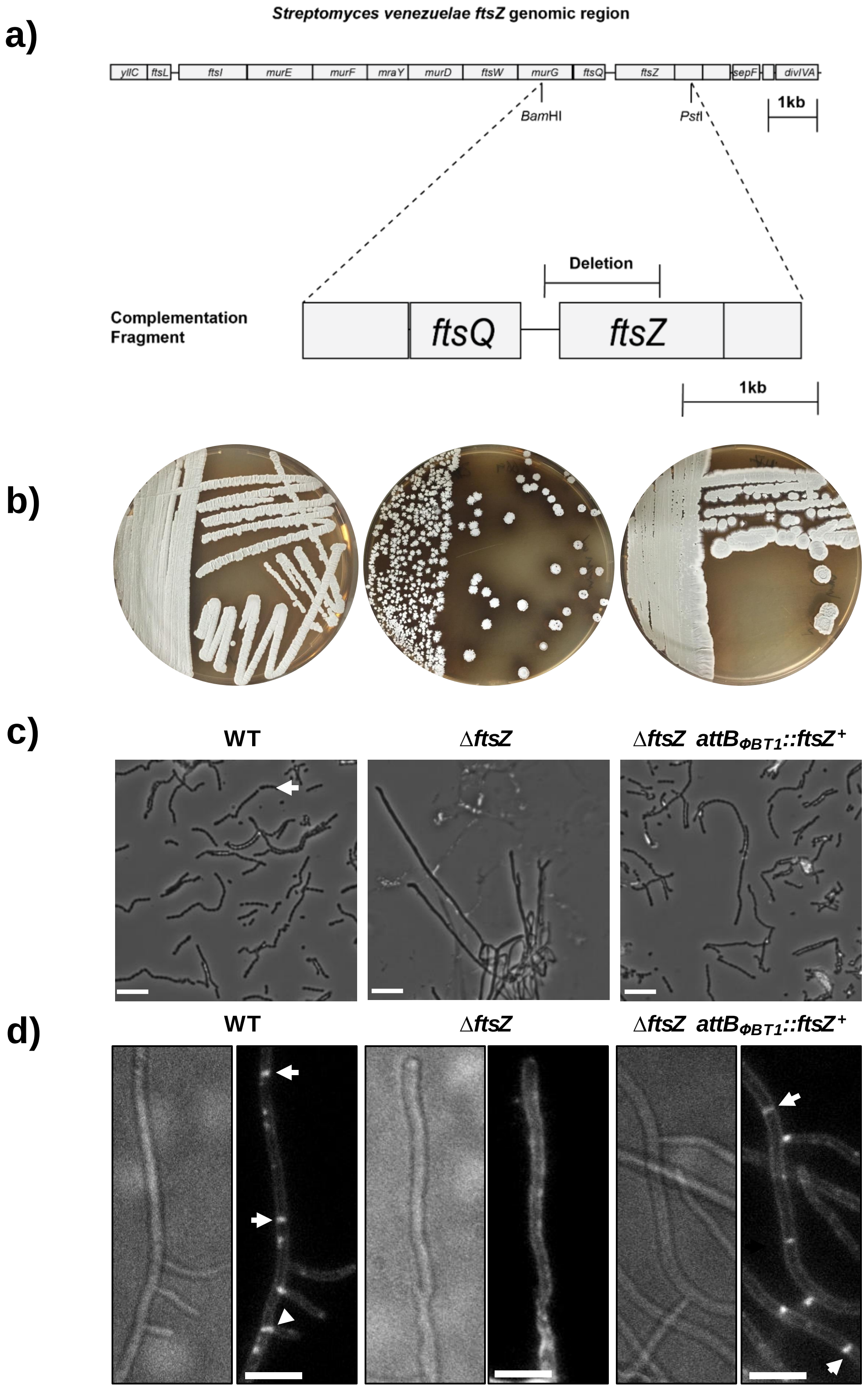

Supplementary Figure 1. Map of the *Streptomyces venezuelae ftsZ* genomic region and comparison of growth and cross wall formation in wild type, Δ*ftsZ* and complemented cells.

a) The region used in the complementation vector is expanded below the gene map. The deleted region of the *ftsZ* open reading frame is indicated above the *ftsZ* gene in the expanded map.

b) Complementation of the *ftsZ*-null insertion deletion mutant. From left to right: wild type *Streptomyces venezuelae*, the *ftsZ*-null mutant (DU669) and the *ftsZ*-null mutant containing the complementation vector, pJS8 (DU670) were grown on MYM agar for 4 days.

c) Impression cover slip lifts were made and analysed by phase contrast microscopy. Scale bar = 5 µm.

d) Light (left) and fluorescence (right) microscopy image pairs of wild type *Streptomyces venezuelae*, the *ftsZ*-null mutant (DU669) and the *ftsZ*-null mutant containing *ftsZ* complementation vector, pJS8 (DU670) grown on MYM agar for 18 hours. Impression coverslip lifts were made and mounted in PBS containing 0.5 µg/ml FM4-64 on 1% agarose pads. Arrows = membrane staining of vegetative cross-walls. Scale bars = 5 µm

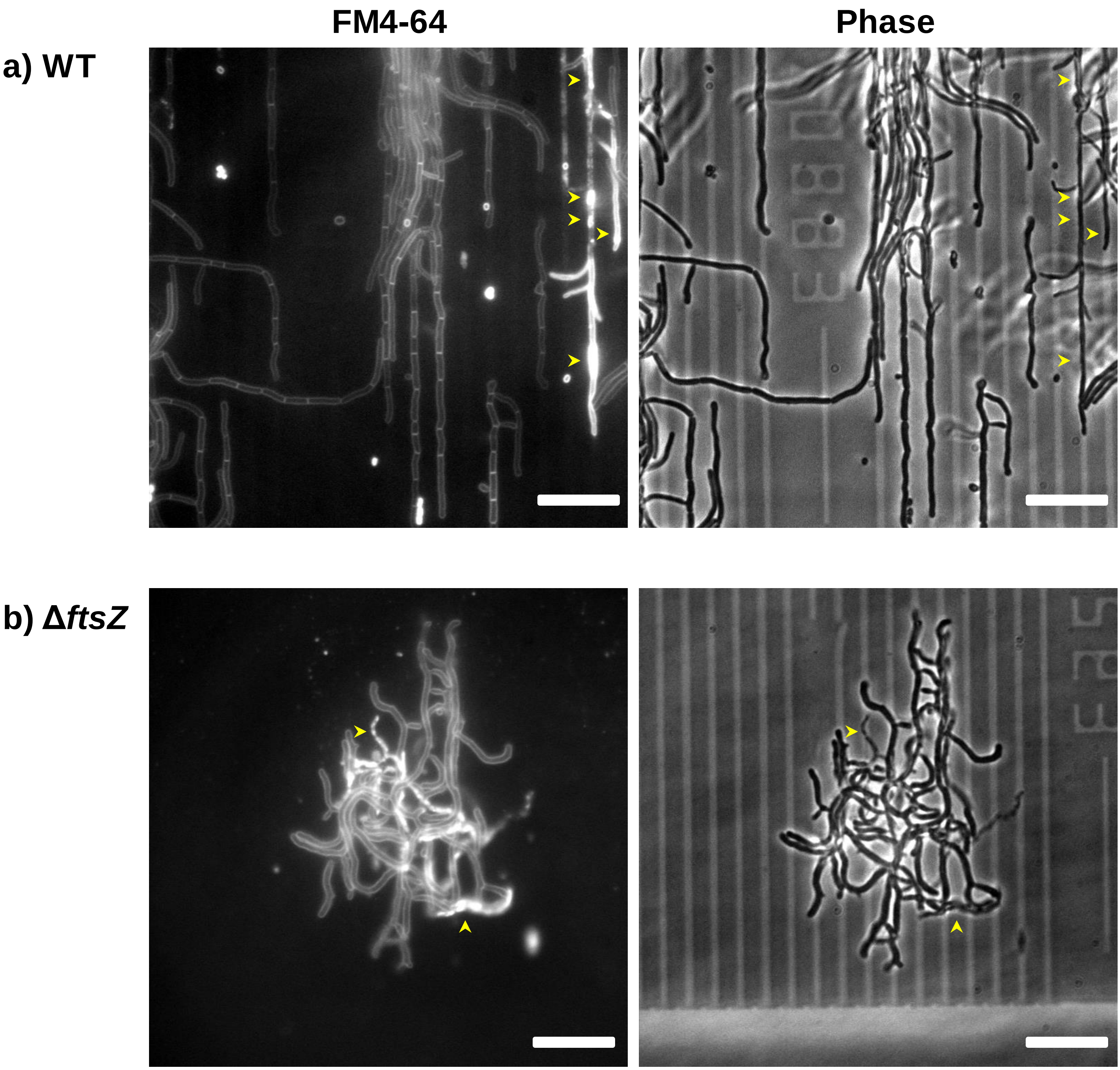

Supplementary Figure 2. Membrane accumulations are evident at several places in the lysed compartments (see yellow arrowheads). The panels show FM4-64 stained membrane images (left) and brightfield images (right) of the wild type (a) and Δ*ftsZ* strains. The cells were grown in GYM medium with FM4-64 dye in our home-made microfluidic device during 18 h. Scale bars = 10 µm.

a) WT

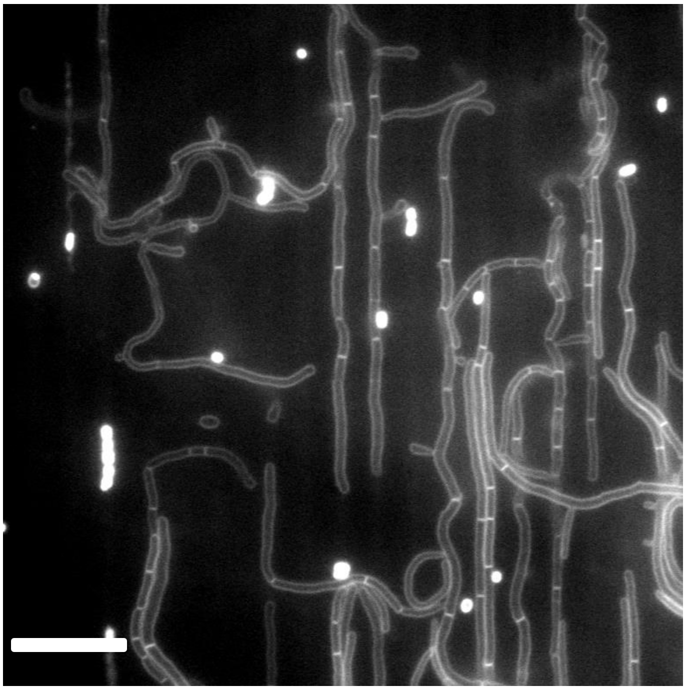

b) Δ*ftsZ*

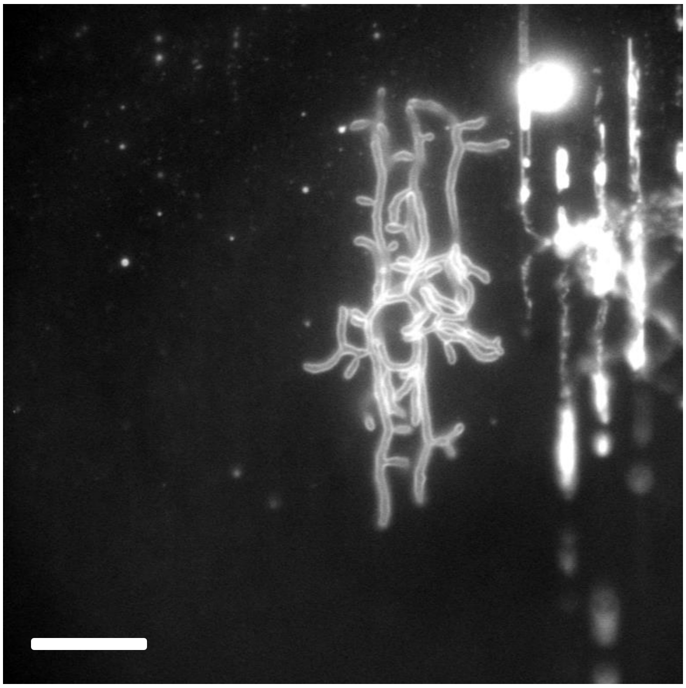

Supplementary Figure 3. Membrane accumulations are not detected in hyphal segments without signs of lysis. The panels show FM4-64 stained membrane images of the wild type (a) and Δ*ftsZ* strains (b). The cells were grown in GYM medium with FM4-64 dye in our home-made microfluidic device during 18 h. Note in the Δ*ftsZ* panels the difference in the staining between the lysed and non-lysed cells. Scale bars = 10 µm.

a) *S. coelicolor* M145 (TSB, FM5-95, Microfluidic device)

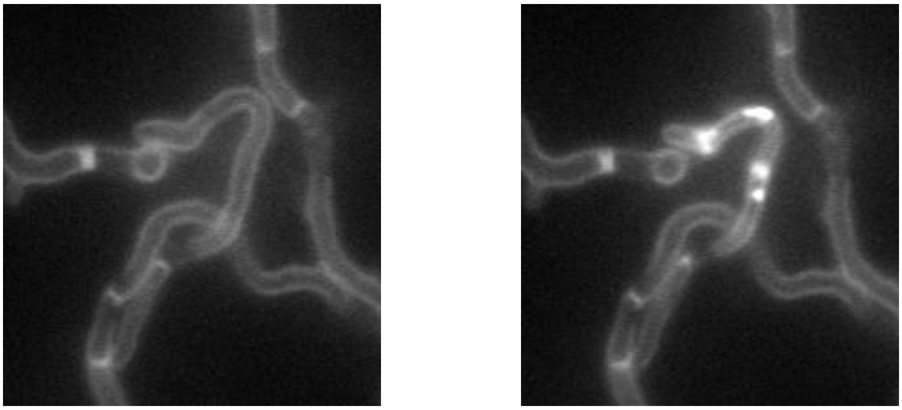

Before lysis **After lysis**

b) *S. coelicolor* M145 (GYM, FM4-64, Flask)

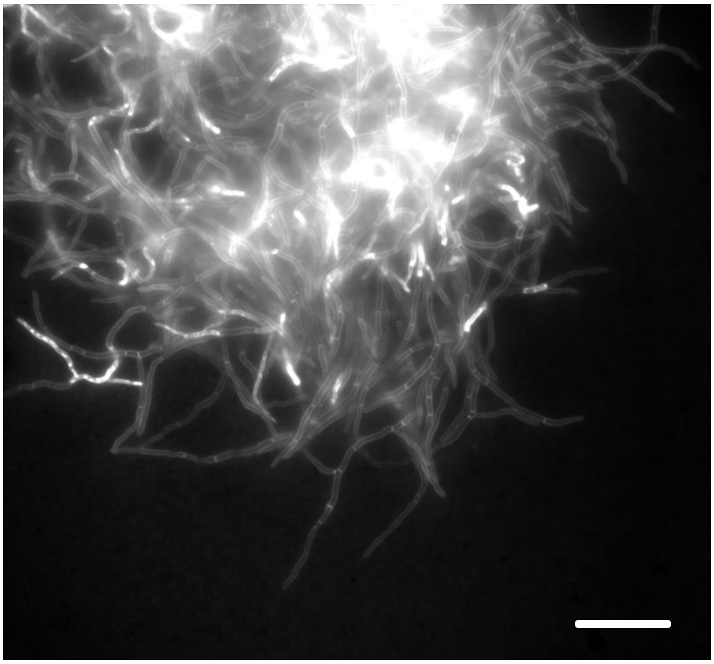

Supplementary Figure 4. Membrane accumulations are not detected in hyphal segments without signs of lysis. a) The panels show FM5-95 stained membrane images (of two sequential frames) of *S. coelicolor* M145 grown in TSB medium using our home-made microfluidic device. **b)** The *S. coelicolor* M145 cells were grown in flasks using GYM medium and stained with FM4-64 dye before observation in the Microscope. Note the different pattern of staining in the distinct compartments. Scale bars = 10 µm.

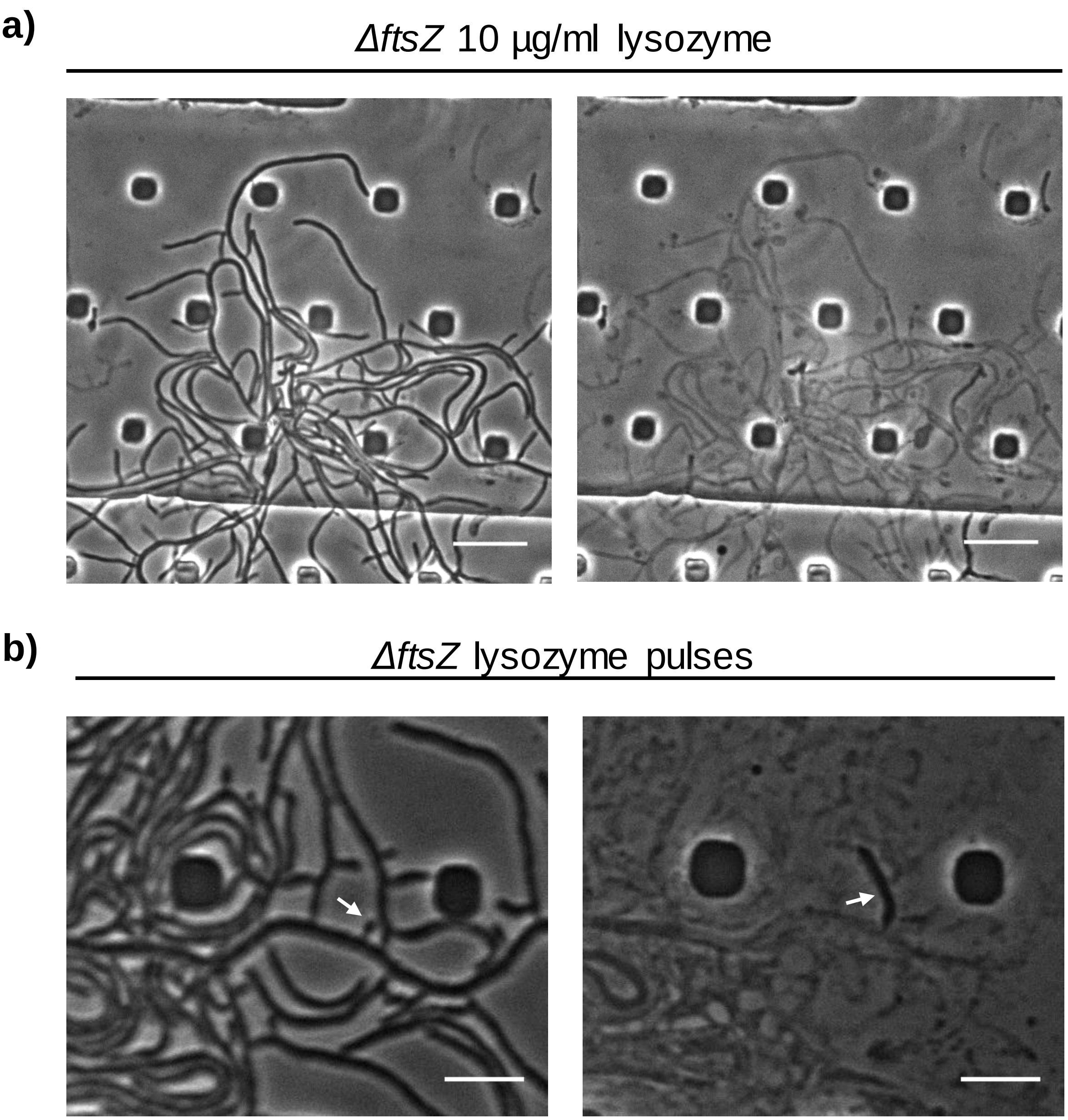

Supplementary Figure 5. Lack of compartmentalisation in the Δ*ftsZ* mutant (survival and sealing may involve branch points). a) Growth of Δ*ftsZ* mutant mycelia in the CellASIC ONIX microfluidic chamber in TSB medium with constant lysozyme flow up to 10 µg/ml. Scale bar = 10 µm. **b)** Effects of lysozyme pulsing on the Δ*ftsZ* mutant in the CellASIC ONIX microfluidic chamber with TSB medium. White arrow indicates survival of an individual branch. Scale bar = 5 µm.

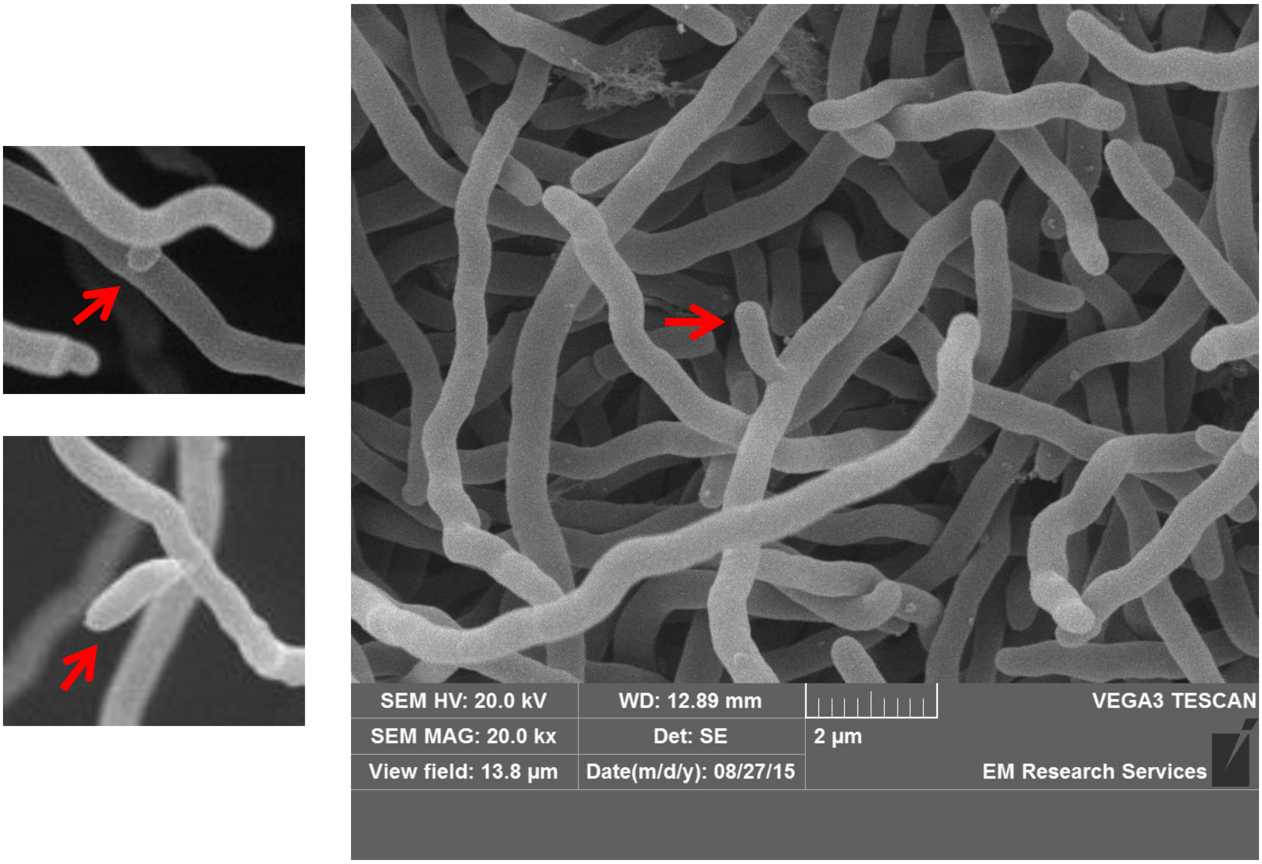

Supplementary Figure 6. Scanning electron microscopy analysis of *S. venezuelae* wild type cells grown in TSA during 4 days. Red arrows point to several branches observed in the analysis.

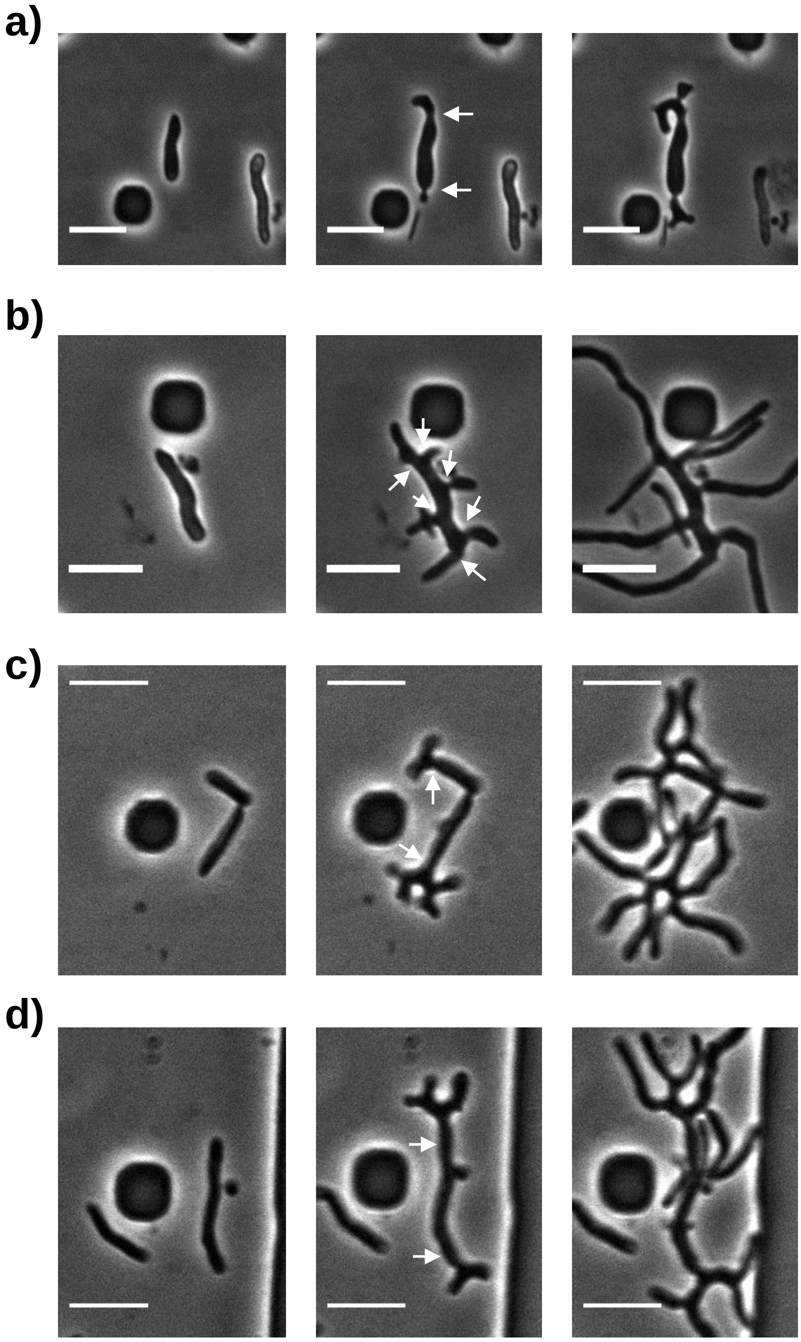

Supplementary Figure 7. Cell re-growth following fragmentation. a-d) representative examples of cell re-growth of fragments of the *ΔftsZ* mutant (a-b) and wild-type (c-d). Fragments were grown in the CellASIC ONIX microfluidic chamber with TSB media. White arrows indicate the initial sites of re-growth. Scale bars = 5 μm.
