# Supplementary material for: A mechanism for FtsZ-independent proliferation in Streptomyces
Source: Nat Commun. 2017 Nov 9;8:1378. doi: 10.1038/s41467-017-01596-z (PMC5680176; doi:10.1038/s41467-017-01596-z)
Supplement: Supplementary file 2 — Description of Additional Supplementary Files [file 41467_2017_1596_MOESM2_ESM.pdf]

Description of Additional Supplementary Files

File Name: Supplementary Movie 1
Description: GYM media was supplemented with 50 µM NADA to stain the cell wall. Images were acquired every 5 min and the movie is displayed at 7 frames per second.

File Name: Supplementary Movie 2
Description: TSB media was supplemented with 50 µM NADA to stain the cell wall. Images were acquired every 5 min and the movie is displayed at 7 frames per second.

File Name: Supplementary Movie 3
Description: Difco Nutrient Broth was supplemented with 50 µM NADA to stain the cell wall. Images were acquired every 5 min and the movie is displayed at 7 frames per second.

File Name: Supplementary Movie 4
Description: Mutant fragments of *S. venezualae* from example 1 were imaged in a homemade microfluidic device in GYM media supplemented with 0.5 µg ml^{-1} FM4-64. Video shows Bright field channel. Cell images were acquired every 15 min and the movie is displayed at 7 frames per second.

File Name: Supplementary Movie 5
Description: Mutant fragments of *S. venezualae* from example 2 were imaged in a homemade microfluidic device in GYM media supplemented with 0.5 µg ml^{-1} FM4-64. Video shows FM4-64 channel. Cell images were acquired every 15 min and the movie is displayed at 7 frames per second.

File Name: Supplementary Movie 6
Description: Mutant fragments of *S. venezualae* from example 3 were imaged in a homemade microfluidic device in GYM media supplemented with 0.5 µg ml^{-1} FM4-64. Video shows Bright field channel. Cell images were acquired every 15 min and the movie is displayed at 7 frames per second.

File Name: Supplementary Movie 7
Description: Mutant fragments of *S. venezualae* from example 4 were imaged in a homemade microfluidic device in GYM media supplemented with 0.5 µg ml^{-1} FM4-64. Video shows FM4-64 channel. Cell images were acquired every 15 min and the movie is displayed at 7 frames per second.

File Name: Supplementary Movie 8
Description: Wild type spores of *S. venezualae* from example 1 were imaged in a homemade microfluidic device in GYM media supplemented with 0.5 µg ml^{-1} FM4-64. Video shows FM4-64 channel. Cell images were acquired every 15 min and the movie is displayed at 7 frames per second.

File Name: Supplementary Movie 9
Description: Wild type spores of *S. venezualae* from example 2 were imaged in a homemade microfluidic device in GYM media supplemented with 0.5 μg ml^{-1} FM4-64. Video shows FM4-64 channel. Cell images were acquired every 15 min and the movie is displayed at 7 frames per second.

File Name: Supplementary Movie 10
Description: Wild type spores of *S. venezualae* from example 3 were imaged in a homemade microfluidic device in GYM media supplemented with 0.5 μg ml^{-1} FM4-64. Video shows FM4-64 channel. Cell images were acquired every 15 min and the movie is displayed at 7 frames per second.

File Name: Supplementary Movie 11
Description: Wild type spores of *S. venezualae* from example 4 were imaged in a homemade microfluidic device in GYM media supplemented with 0.5 μg ml^{-1} FM4-64. Video shows FM4-64 channel. Cell images were acquired every 15 min and the movie is displayed at 7 frames per second.

File Name: Supplementary Movie 12
Description: Wild type spores of *S. coelicolor* from example 1 were imaged in a homemade microfluidic device in TSB media supplemented with 0.5 μg ml^{-1} FM5-95. Video shows FM5-95 channel. Cell images were acquired every 15 min and the movie is displayed at 7 frames per second.

File Name: Supplementary Movie 13
Description: Wild type spores of *S. coelicolor* from example 2 were imaged in a homemade microfluidic device in TSB media supplemented with 0.5 μg ml^{-1} FM5-95. Video shows FM5-95 channel. Cell images were acquired every 15 min and the movie is displayed at 7 frames per second.

File Name: Supplementary Movie 14
Description: Wild type spores of *S. venezualae* from example 1 were imaged in the CellASIC ONIX device in TSB media in the continuous presence of lysozyme up to 1000 μg ml^{-1}. Cell images were acquired every 5 min and the movie is displayed at 7 frames per second.

File Name: Supplementary Movie 15
Description: Wild type spores of *S. venezualae* from example 2 were imaged in the CellASIC ONIX device in TSB media in the continuous presence of lysozyme up to 1000 μg ml^{-1}. Cell images were acquired every 5 min and the movie is displayed at 7 frames per second.

File Name: Supplementary Movie 16
Description: Fragments of the *S. venezualae ΔftsZ* mutant from example 1 were imaged in the CellASIC ONIX device in TSB media in the continuous presence of lysozyme up to 1000 μg ml^{-1}. Cell images were acquired every 5 min and the movie is displayed at 7 frames per second.

File Name: Supplementary Movie 17
Description: Fragments of the *S. venezualae ΔftsZ* mutant from example 2 were imaged in the CellASIC ONIX device in TSB media in the continuous presence of lysozyme up to 100 µg ml^{-1}. Cell images were acquired every 5 min and the movie is displayed at 7 frames per second.

File Name: Supplementary Movie 18
Description: Fragments of the *S. venezualae ΔftsZ* mutant from example 3 were imaged in the CellASIC ONIX device in TSB media in the continuous presence of lysozyme up to 10 µg ml^{-1}. Cell images were acquired every 5 min and the movie is displayed at 7 frames per second.

File Name: Supplementary Movie 19
Description: Fragments of the *S. venezualae ΔftsZ* mutant from example 4 were imaged in the CellASIC ONIX device in TSB media in the continuous presence of lysozyme up to 10 µg ml^{-1}. Cell images were acquired every 5 min and the movie is displayed at 7 frames per second.

File Name: Supplementary Movie 20
Description: Fragments of the *S. venezualae ΔftsZ* mutant from example 1 were imaged in the CellASIC ONIX device in the presence of TSB media. Four pulses of lysozyme (two at 10 µg ml^{-1}, two at 100 µg ml^{-1}) were given to the cells during the time lapse. A clear example of fragment survival is shown. Images were acquired every 5 min and the movie is displayed at 7 frames per second.

File Name: Supplementary Movie 21
Description: Fragments of the *S. venezualae ΔftsZ* mutant from example 1 were imaged in the CellASIC ONIX device in the presence of TSB media. Four pulses of lysozyme (two at 10 µg ml^{-1}, two at 100 µg ml^{-1}) were given to the cells during the time lapse. The movie shows the whole field view of fragment survival seen in Supplementary Movie 20. Images were acquired every 5 min and the movie is displayed at 7 frames per second.

File Name: Supplementary Movie 22
Description: Fragments of the *S. venezualae ΔftsZ* mutant from example 2 were imaged in the CellASIC ONIX device in the presence of TSB media. Four pulses of lysozyme (two at 10 µg ml^{-1}, two at 100 µg ml^{-1}) were given to the cells during the time lapse. Images were acquired every 5 min and the movie is displayed at 7 frames per second.

File Name: Supplementary Movie 23
Description: Fragments of the *S. venezualae ΔftsZ* mutant from example 3 were imaged in the CellASIC ONIX device in the presence of TSB media. Four pulses of lysozyme (two at 10 µg ml^{-1}, two at 100 µg ml^{-1}) were given to the cells during the time lapse. Images were acquired every 5 min and the movie is displayed at 7 frames per second.

File Name: Supplementary Movie 24
Description: Fragments of the *S. venezualae ΔftsZ* mutant from example 4 were imaged in the CellASIC ONIX device in the presence of TSB media. Four pulses of lysozyme (two at 10 μg ml^{-1}, two at 100 μg ml^{-1}) were given to the cells during the time lapse. A clear example of fragment survival is shown. Images were acquired every 5 min and the movie is displayed at 7 frames per second.

File Name: Supplementary Movie 25
Description: Fragments of the *S. venezualae ΔftsZ* mutant from example 5 were imaged in the CellASIC ONIX device in the presence of TSB media. Four pulses of lysozyme (two at 10 μg ml^{-1}, two at 100 μg ml^{-1}) were given to the cells during the time lapse. Images were acquired every 5 min and the movie is displayed at 7 frames per second.

File Name: Supplementary Movie 26
Description: Fragments of the *S. venezualae ΔftsZ* mutant from example 6 were imaged in the CellASIC ONIX device in the presence of TSB media. Four pulses of lysozyme (two at 10 μg ml^{-1}, two at 100 μg ml^{-1}) were given to the cells during the time lapse. A clear example of fragment survival is shown. Images were acquired every 5 min and the movie is displayed at 7 frames per second.

File Name: Supplementary Movie 27
Description: Wild type spores from example 1 were grown in the CellASIC ONIX device in the presence of TSB media. Four pulses of lysozyme (two at 10 μg ml^{-1}, two at 100 μg ml^{-1}) were given to the cells during the time lapse. Images were acquired every 5 min and the movie is displayed at 7 frames per second.

File Name: Supplementary Movie 28
Description: Wild type spores from example 2 were grown in the CellASIC ONIX device in the presence of TSB media. Four pulses of lysozyme (two at 10 μg ml^{-1}, two at 100 μg ml^{-1}) were given to the cells during the time lapse. Images were acquired every 5 min and the movie is displayed at 7 frames per second.

File Name: Supplementary Movie 29
Description: Wild type spores from example 3 were grown in the CellASIC ONIX device in the presence of TSB media. Four pulses of lysozyme (two at 10 μg ml^{-1}, two at 100 μg ml^{-1}) were given to the cells during the time lapse. Images were acquired every 5 min and the movie is displayed at 7 frames per second.

File Name: Supplementary Movie 30
Description: Wild type spores from example 4 were grown in the CellASIC ONIX device in the presence of TSB media. Four pulses of lysozyme (two at 10 μg ml^{-1}, two at 100 μg ml^{-1}) were given to the cells during the time lapse. Movie shows survival of the wild type at a branch point. Images were acquired every 5 min and the movie is displayed at 7 frames per second.

File Name: Supplementary Movie 31
Description: Wild type spores from example 5 were grown in the CellASIC ONIX device in the presence of TSB media. Four pulses of lysozyme (two at 10 µg ml^{-1}, two at 100 µg ml^{-1}) were given to the cells during the time lapse. Movie shows survival of the wild type at a branch point. Images were acquired every 5 min and the movie is displayed at 7 frames per second.

File Name: Supplementary Movie 32
Description: Representative example of cell re-growth of fragments of the *ΔftsZ* mutant showing growth from both poles or premature lysis. Fragments were grown in the CellASIC ONIX microfluidic chamber with TSB media. Images were acquired every 5 mins and the movie is displayed at 7 frames per second.

File Name: Supplementary Movie 33
Description: Representative example of cell re-growth of fragments of the *ΔftsZ* mutant showing growth from multiple sites. Fragments were grown in the CellASIC ONIX microfluidic chamber with TSB media. Images were acquired every 5 mins and the movie is displayed at 7 frames per second.

File Name: Supplementary Movie 34
Description: Representative example of cell re-growth of fragments of the *ΔftsZ* mutant showing growth with an undefined pattern. Fragments were grown in the CellASIC ONIX microfluidic chamber with TSB media. Images were acquired every 5 mins and the movie is displayed at 7 frames per second.

File Name: Supplementary Movie 35
Description: Representative example of cell re-growth of fragments of wild type showing growth with an undefined pattern. Fragments were grown in the CellASIC ONIX microfluidic chamber with TSB media. Images were acquired every 5 mins and the movie is displayed at 7 frames per second.

File Name: Supplementary Movie 36
Description: Representative example of cell re-growth of from wild type spores showing DivIVA tip localisation. Cells were grown in the CellASIC ONIX microfluidic chamber with TSB media. Images were acquired every 15 mins and the movie is displayed at 4 frames per second.

File Name: Supplementary Movie 37
Description: Representative example of DivIVA tip localisation in *ΔftsZ* mutant fragments. Cells were grown in the CellASIC ONIX microfluidic chamber with TSB media. Images were acquired every 15 mins and the movie is displayed at 4 frames per second

File Name: Supplementary Movie 38
Description: Representative example of DivIVA tip localisation in *ΔftsZ* mutant fragments from example 2 (containing DivIVA-mCherry) grown in the CellASIC ONIX microfluidic chamber with TSB media showing growth from both poles. Images were acquired every 15 mins and the movie is displayed at 1 frame per second.

File Name: Supplementary Movie 39
Description: Representative example of DivIVA tip localisation in *ΔftsZ* mutant fragments from example 3 (containing DivIVA-mCherry) grown in the CellASIC ONIX microfluidic chamber with TSB media showing growth from multiple sites. Images were acquired every 15 mins and the movie is displayed at 1 frame per second.

File Name: Supplementary Movie 40
Description: Representative example of DivIVA tip localisation in *ΔftsZ* mutant fragments from example 4 (containing DivIVA-mCherry) grown in the CellASIC ONIX microfluidic chamber with TSB media showing growth with a bulging-like pattern. Images were acquired every 15 mins and the movie is displayed at 1 frame per second.

File Name: Supplementary Movie 41
Description: Fragments of the *ΔftsZ* mutant (containing DivIVA-mCherry) were grown in the CellASIC ONIX microfluidic chamber with TSB media and subject to four pulses of lysozyme (two at 10 µg ml^{-1}, two at 100 µg ml^{-1}) during the time lapse. Images were acquired every 5 min and the movie is displayed at 7 frames per second.
